# Intraventricular neuroepithelial tumors: surgical outcome, technical considerations and review of literature

**DOI:** 10.1186/s12885-020-07570-1

**Published:** 2020-11-03

**Authors:** A. Kaywan Aftahy, Melanie Barz, Philipp Krauss, Friederike Liesche, Benedikt Wiestler, Stephanie E. Combs, Christoph Straube, Bernhard Meyer, Jens Gempt

**Affiliations:** 1 Department of Neurosurgery, Technical University Munich, Medical Faculty, School of Medicine, Klinikum rechts der Isar, Ismaninger Str. 22, 81675 Munich, Germany; 2 Department of Neuropathology, Technical University Munich, School of Medicine, Klinikum rechts der Isar, Institute of Pathology, Munich, Germany; 3 Department of Neuroradiology, Technical University Munich, School of Medicine, Klinikum rechts der Isar, Munich, Germany; 4 Department of Radiation Oncology, Technical University Munich, School of Medicine, Klinikum rechts der Isar, Munich, Germany; 5 Department of Radiation Sciences (DRS) Helmholtz Zentrum Munich, Institute of Innovative Radiotherapy (iRT), Munich, Germany; 6 German Cancer Consortium (DKTK), Partner Site Munich, Munich, Germany

**Keywords:** Intraventricular tumor, Neuroepithelial, Ependymoma, Subependymoma, Central neurocytoma, Surgical technique, Extent of resection, Neurosurgery

## Abstract

**Background:**

Intraventricular neuroepithelial tumors (IVT) are rare lesions and comprise different pathological entities such as ependymomas, subependymomas and central neurocytomas. The treatment of choice is neurosurgical resection, which can be challenging due to their intraventricular location. Different surgical approaches to the ventricles are described. Here we report a large series of IVTs, its postoperative outcome at a single tertiary center and discuss suitable surgical approaches.

**Methods:**

We performed a retrospective chart review at a single tertiary neurosurgical center between 03/2009–05/2019. We included patients that underwent resection of an IVT emphasizing on surgical approach, extent of resection, clinical outcome and postoperative complications.

**Results:**

Forty five IVTs were resected from 03/2009 to 05/2019, 13 ependymomas, 21 subependymomas, 10 central neurocytomas and one glioependymal cyst. Median age was 52,5 years with 55.6% (25) male and 44.4% (20) female patients. Gross total resection was achieved in 93.3% (42/45). 84.6% (11/13) of ependymomas, 100% (12/21) of subependymomas, 90% (9/10) of central neurocytomas and one glioependymal cyst were completely removed. Postoperative rate of new neurological deficits was 26.6% (12/45). Postoperative new permanent cranial nerve deficits occurred in one case with 4th ventricle subependymoma and one in 4th ventricle ependymoma. Postoperative KPSS was 90% (IR 80–100). 31.1% of the patients improved in KPSS, 48.9% remained unchanged and 20% declined. Postoperative adverse events rate was 20.0%. Surgery-related mortality was 2.2%. The rate of shunt/cisternostomy-dependent hydrocephalus was 13.3% (6/45). 15.4% of resected ependymomas underwent adjuvant radiotherapy. Mean follow-up was 26,9 (±30.1) months.

**Conclusion:**

Our surgical findings emphasize satisfactory complete resection throughout all entities. Surgical treatment can remain feasible, if institutional experience is given. Satisfying long-term survival and cure is possible by complete removal. Gross total resection should always be performed under function-remaining aspects due to mostly benign or slow growing nature of IVTs. Further data is needed to evaluate standard of care and alternative therapy options in rare cases of tumor recurrence or in case of patient collective not suitable for operative resection.

## Background

Intraventricular neuroepithelial tumors (IVT) are rare lesions and account for 2–7% of intracranial tumors [1]. IVTs summarize a group of different pathological entities, namely ependymoma, subependymoma, central neurocytoma and glioependymal cysts. These lesions are mainly benign and arise from the ventricular wall or the choroid plexus [2–4]. Due to their benign character and ventricular location, they first become apparent by signs of hydrocephalus or are incidental findings. As IVTs are regularly not targetable by radiation or systemic therapy, until now, surgical resection presents the treatment of choice.

One of the first successful intraventricular resections were performed by Krause in 1913 by an infratentorial supracerebellar approach [5]. Jamieson’s [6] and Poppen’s [7] occipital trans-tentorial, and Dandy’s posterior transcallosal approach [8] were further landmark techniques for entering the ventricular system.

Over the last decades, further authors have proposed technically advantaged approaches to the ventricular system. Regarding the fourth ventricle, approaches as the transvermian [9], a subtonsillar-transcerebellomedullary [10], a superior transvelar approach [11] or also endoscopic approaches [12, 13] were described with claiming to be the superior one. The transvermian approach has been performed frequently in history, but data showed high rates of cerebellar mutism and disequilibrium [14, 15]. To enter the lateral and/or third ventricle a variety of approaches including the frontal-transcortical, the anterior/posterior interhemispheric-transcallosal and the contralateral interhemispheric-transfalcine-transprecuneal approach have been described [16–21].

Due to the above-mentioned diversity, aim of this manuscript is to share our experience with a large series of IVTs at a single tertiary neurosurgical center by using technical acceptable and standardized approaches to the ventricle system. With focus on few, but well-experienced approaches this study also want to show their sufficiency and reduction of perioperative morbidity. Furthermore, due to the rare natural history of IVTs, the heterogeneity was chosen to discuss this study from a surgical and technical point of view.

## Methods

### Study design and outcome parameters

We performed an observational retrospective single-center study. Adult patients who underwent surgery for IVT between March 2009 and May 2019 were included. The clinical records of patients were analyzed according to surgical approach, pre- and postoperative neurological/ophthalmological status, Karnofsky Performance Status Scale (KPSS) and adverse events according to the Clavien Dindo scale (CDG) during follow up visits. Extent of resection was determined by pre- and postoperative T1 ± contrast agent 3.0 T MRI images. Infant patients and patients not undergoing surgery were excluded. All surgeries were performed under general anesthesia by experienced neurooncological surgeons in our neurosurgical institute. For details on the surgical approach see discussion/surgical approach.

### Statistical analysis

Statistical analysis, including descriptive data analysis, was performed using IBM SPSS Statistics Version 26.0 (SPSS Inc., IBM Corp., Armonk, NY, USA). Normal distribution was assumed according to the central limit theorem. Data in text and graphs are shown as median with interquartile range (IQR) or mean ± standard deviation (SD). Survival analyses were performed using Kaplan-Meier estimates for univariate analysis and Cox regression proportional hazards model for multivariate analysis. A *p* value ≤ .05 was considered significant and indicated by “*”, *p* values ≤ .01 were indicated by “**”, and values ≤ .001 by “***”.

#### Ethics approval and consent to participate

Our study was approved by the local ethics committee, Technical University Munich, School of Medicine, (N°5625–12). It is conducted in accordance with the ethical standards of the 1964 Declaration of Helsinki and its later amendments [22]. The requirement for written informed consent was waived by the ethics committee.

## Results

### Patient population

Forty-five patients with IVT underwent surgical resection between March 2009 and May 2019. Median age was 52,5 years with 55.6% (25) male and 44.4% (20) female patients. 15.6% (7/45) of the patients were asymptomatic, tumors were discovered incidentally. 84.4% 38/45) were symptomatic–including cephalgia, nausea, diplopia, cranial nerve deficits, ataxia/imbalance and further symptoms. The median preoperative KPSS was 90% (IR 90–90) and the median postoperative KPSS was 90% (IR 80–100). No evidence of spinal drop metastasis was present on preoperative (holo-spinal) MR imaging (Table 1).

**Table 1:** Demographics and preoperative characteristics

Demographics % (N) or mean/median (SD/IR)	Ependymoma (13)	Subependymoma (21)	Central Neurocytoma (10)	GEC (1)	Total (45)
Age	54.7 (±20.2)	58 (±12)	41.7 (±15.2)	18	52.5 (±17.1)
Sex	M 46.2% (6)	M 61.9% (13)	M 60.0% (6)	M 0	M 25 (55.6%)
	F 53.8% (7)	F 38.1% (8)	F 40.0% (4)	F 1	F 20 (44.4%)
Clinical presentation					
Pre-operative KPSS	90% (IR 80–90)	90% (IR 90–90)	90 (IR 90–90)	90	90 (IR90–90)
Asymptomatic	30.8% (4)	9.6% (2)	10.0% (1)	0	15.6% (7)
Recurrence	15.4% (2)	0	10.0% (1)		6.6% (3)
Cranial nerve deficits	III 7.7% (1) V 7.7% (1) IX 15.4% (2) XII 7.7% (1)	0	0	0	11.1% (5)
Diplopia	7.7% (1)	4.8% (1)	10.0% (1)	0	6.7% (3)
Cephalgia	38.5% (5)	42.9% (9)	80.0% (8)	0	48.9% (22)
Vertigo/Nausea	0	47.6% (10)	50.0% (5)	0	33.3% (15)
Hydrocephalus / Concentration disorder	23.1% (3)	23.8% (5)	20.0% (2)	100% (1)	24.4% (11)
Disequilibrium / Ataxia / Cerebellar symptoms	30.8% (4)	9.5% (2)	0	0	13.3% (6)
Dysphagia / Dysarthria	7.7% (1)	0	0	0	2.2% (1)
Hemihypesthesia	7.7% (1)	0	0	0	2.2% (1)

### Tumor related findings and location

Histopathological analysis revealed ependymoma in 13 cases, subependymoma in 21 cases, neurocytomas in 10 cases and glioependymal cyst (GEC) in one case, confirmed by histopathological examination as well. 44.4% (20/45) underwent a frontal-transcortical-keyhole, 48.9% (22/45) a median suboccipital telovelar, 2.2% (1/45) a frontotemporal, further 2.2% (1/45) a supracerebellar-infratentorial and another 2.2% (1/45) a parietal transcortical approach. In three patients with 4th ventricle tumors, which extended more caudally, a C1 arch resection was performed additionally.

92.3% (12/13) of the ependymomas were located in the 4th ventricle, one ependymoma was located in the left temporal horn. 52.4% (11/21) of the subependymomas were located in the lateral ventricles, 47.6% (10/21) in the fourth ventricle. 90.0% (9/10) of the central neurocytomas were found in lateral ventricles (three right, five left), one was located at the floor of the 3rd ventricle. The only GEC, which caused an occlusive hydrocephalus, was located at the roof of the 3rd ventricle (Table 2).

**Table 2:** Tumor entity, WHO grade and intraventricular location

Tumor % (N) or mean/median (SD/IR)	N	WHO	Ventricle location	Location within			Approach according to location
				Lateral ventricles	3rd	4th	
Ependymoma	13	II (12) III (1)	Lateral ventricle 7.7% (1)	Left temporal horn (1)			Frontotemporal (1)
			4th ventricle 92.3% (12)			Below str. Med 100% (12)	TeloVelar (12)
Subependymoma	21	I II (1)	Lateral ventricle 52.4% (11)	Left frontal horn 36.4% (4) Right frontal horn 63.6% (7)			Frontal-Keyhole (10) Parietal craniotomy (1)
			4th ventricle 47.6% (10)			Below str. Med. 90.0% (9) Above str. Med. 10.0% (1)	TeloVelar (10)
Central Neurocytoma	10	II	Lateral ventricle 90.0% (9)	Left frontal horn 55.6% (5) Right frontal horn 44.4% (4)			Frontal-Keyhole (9)
			3rd ventricle 10.0% (1)		Floor (1)		Infratententorial-supracerebellar (1)
GEC	1	–	3rd ventricle 100% (1)		Roof (1)		Frontal-Keyhole (1)
Total	45		Lateral ventricle 46.7% (21) 3rd ventricle 4.4% (2) 4th ventricle 48.9% (22)				Frontal-Keyhole 44.4% (20) Others 6.7% (3) TeloVelar 48.9% (22)

In total, 92.3% (12/13) of the ependymomas and 47.6% (10/21) of the subependymomas were located infratentorial (4th ventricle) but none of the central neurocytomas or the GEC. In sum, 48.9% (22/45) of all analyzes tumors were located in the 4th ventricle. 4.4% (2/45) of the tumors were located in the 3rd ventricle, one central neurocytoma and the GEC. 46.7% (21/45) of the tumors were found in the lateral ventricles (1/13 ependymomas, 11/21 subependymomas, 9/10 central neurocytomas).

### Functional outcome and surgical complications

Complete removal could be achieved in 93.3% (42/45) (Table 3). No statistically significant predictive factors regarding overall survival could be analyzed in a univariate analysis. 2.6% (12/45) developed new postoperative neurological deficits, 58.3% were permanent during follow-up. 20.0% (2/10) of patients with central neurocytoma presented with a latent hemiparesis and 20.0% (2/10) with transient dysarthria. One patient with a 4th ventricle ependymoma developed postoperative hemorrhagic infarction with KPSS decline from 80.0 to 40.0%. This patient also developed a shunt-dependent hydrocephalus. One patient with 4th ventricle subependymoma died due to central lung artery embolism postoperatively. 13.3% (6/45) of the patients had postoperative ventriculitis/meningitis (2/21 subependymomas and 4/10 central neurocytomas), four were aseptic and two (both central neurocytomas) had positive proof of germs. Another patient with a central neurocytoma developed a postoperative ventricle entrapment and underwent cisternostomy. Adverse event rate was 20.0% (9/45). Postoperative KPSS was 90.0% (IR 80–100), 31,1% (14/45) showed an improvement of KPSS (two ependymomas, nine subependymomas, two central neurocytoma and one GEC), but also 20.0% (9/45) declined. The Clavien Dindo Scale (CDG) for postoperative adverse events showed satisfying postoperative outcomes through all entities (1, IR 1–2). Two resected WHO grade II ependymomas underwent adjuvant radiotherapy (15,4%). Mean follow-up was 26.9 months (0–120 months).

**Table 3:** Postoperative clinical characteristics, complications and outcome

Postoperative presentation % (N) or mean/median (SD/IR)	Ependymoma (13)	Subependymoma (21)	Central Neurocytoma (10)	GEC (1)	Total (45)
Gross total resection	84.6% (11)	100% (21)	90.0% (9)	100% (1/1)	93.3% (29)
New neurological deficits	Vigilance 7,.% (1) Ataxia 15.4% (2)	Ataxia 14.3% (3)	Hemiparesis 20.0% (2) Dysarthria 20.0% (2) Tinnitus 10.0% (1) Vigilance 10.0% (1)	0	26.6% (12)
New cranial nerve deficits	IX 7.7% (1) XII 7.7% (1)	VII 4.8% (1) IX 4.8% (1) XII 4.8% (1)	0	0	11.1% (5)
Post-operative KPSS	90% (IR 70–100)	100% (IR 85–100)	90% (IR 87,5–100)	100%	90% (IR 80–100)
KPSS unchanged	61.5% (8)	38.1% (8)	60.0% (6)	0	48.9% (22)
KPSS declined	23.1% (3)	19.0% (4)	20.0% (2)	0	20.0% (9)
KPSS improved	15.4% (2)	42.9% (9)	20.0% (2)	100% (1)	31.1% (14)
Clavien Dindo Scale (CDG)	1 (IR 1–1,25)	1 (±IR 1–2)	2 (IR 1–3)	1	1 (IR 1–2)
Complications	CSF leakage 7.7% (1)	Ventriculitis/Meningitis 9.5% (2) Death 4.8% (1)	Ventriculitis/ Meningitis 40.0% (4) Ventr. entrapment 10.0% (1)	0	20.0% (9)
Shunt/cisternostomy dependency	15.4% (2)	4.8% (1)	30.0% (3)	0	13.3% (6)
Follow-up time in months	16.4 (±14,1)	316 (±33)	26,.4 (±36,9)	72	26,9 (±30.1)

## Discussion

In our study we report a large single institution series of patients undergoing microsurgical resection of IVT. In a majority of cases, complete resection without neurological deficit could be achieved. A meticulous surgical planning and detailed anatomic knowledge is crucial for successful treatment. One has to separate approaches to the lateral and third ventricle from approaches to infratentorial lesions and the fourth ventricle.

### Surgical approach

#### Approaches to the lateral and third ventricle

To enter the lateral and third ventricle a variety of approaches have been described including the frontal-transcortical, the anterior/posterior interhemispheric-transcallosal and the contralateral interhemispheric-transfalcine-transprecuneal approach [16–21]. They allow excellent visualization of important anatomical structures like the thalamostriatal, anterior-septal and caudate veins, foramen of Monro and choroid plexus [23]. The transcortical approach is supposed to be associated with higher incidence of postoperative seizures and possible neurological deficits due to frontal lobe corticotomy and retraction of the supplemental motor or premotor area, but offers greater access and overview, especially in larger intraventricular lesions. The transcallosal approach is technically more demanding for proper dissection but is supposed to leave more cortical structures intact. However, transcallosal approach is also associated with higher morbidity and, in case of e.g. permanent damage of corpus callosum, with postoperative severe neurological deficits like the well-known “disconnection syndrome” [21, 23]. Taking these circumstances into consideration, the transcortical approach is preferred as a workhorse approach in our institute. Historically, high morbidity ratio in the literature initially led to disqualify the transcortical approach to the lateral and third ventricle at the beginning, but closer review of above-mentioned studies display that extended craniotomies and cortical exposure with rough retraction were transacted. Keyhole exposures are pushing minimal invasive philosophy of modern neurosurgery forward and are associated with less brain damage, comparable to injury caused by ventricle puncture, and offers a much more comfortable approach to the ventricle system [24].

**Fig. 1:**
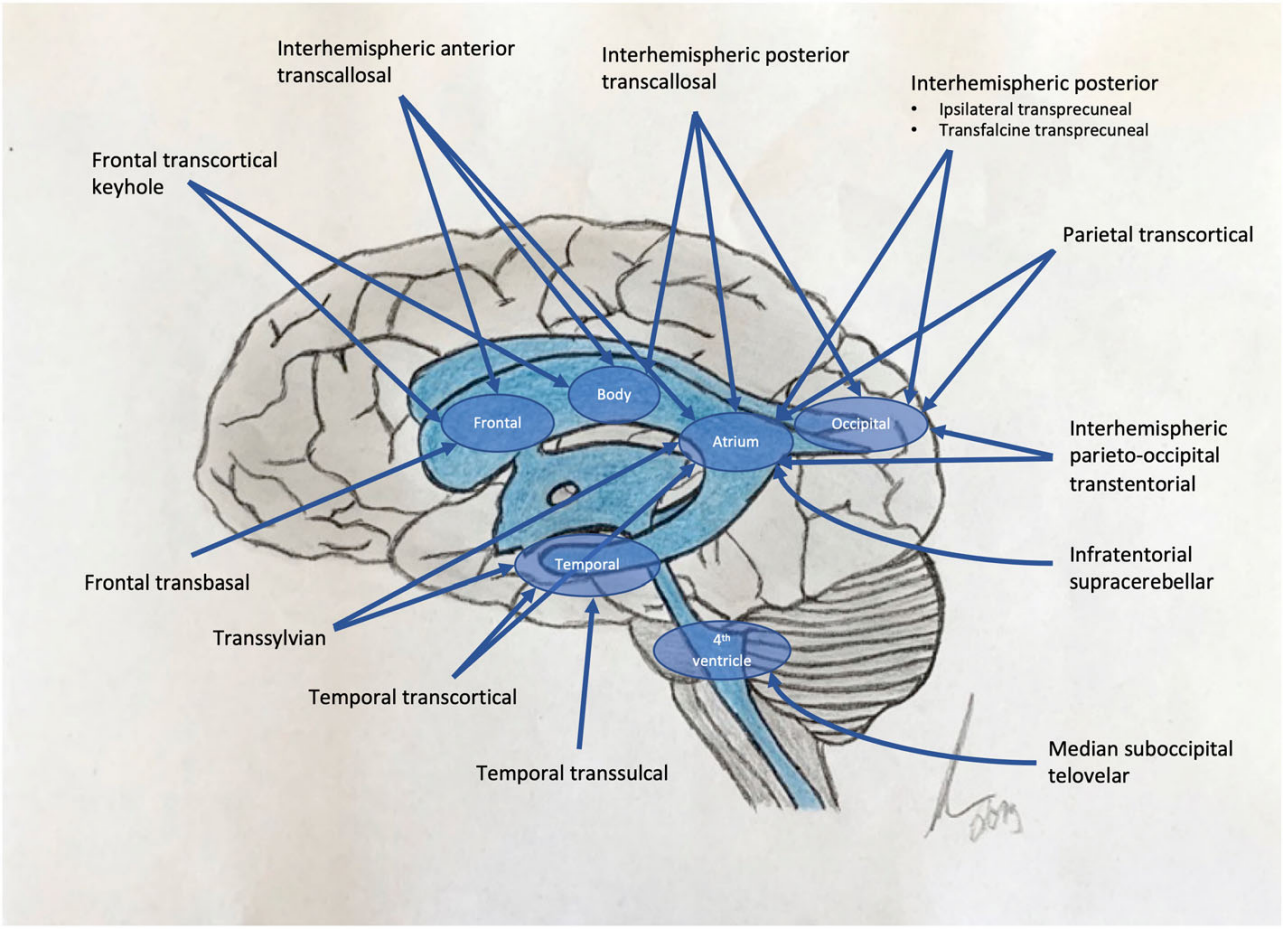
Trajectories and approaches to the lateral and third ventricle. Lateral and third ventricle are shown in blue. Red arrows display the trajectory of the approaches and the parts of the ventricular system reached by that individual approach

Special attention has to be given to the head positioning to ensure optimal conditions. We perform surgery in supine position with ~ 30° anteroflection to elevate the preconorary part of the frontal lobe to the highest point and therefore minimize outflow of cerebrospinal fluid. This avoids collapsing ventricles and tearing of bridging veins. The craniotomy is usually centered on the coronary suture, a diameter of ~ 3 cm is regularly sufficient to guarantee an adequate working canal. Figure 1 summarizes and reflects most prominent approaches.

#### Approaches to the fourth ventricle

To access the fourth ventricle, historically the trans-vermian approach was very popular. Still, this approach harbors the risk of cerebellar mutism and disequilibrium [14, 15] leading to the development of the less invasive median suboccipital telovelar approach [25, 26].

Nevertheless, surgical morbidity of tumors of the fourth ventricle, mostly ependymomas, remains high with up to 30% adverse events. This is probably due to adhesive nature of the lesion and proximity of cranial nerves and their nuclei [14, 27, 28]. To reduce the risk for cranial nerve lesions, monitoring of cranial nerves and electrical intraoperative mapping of the floor of the fourth ventricle should be performed [29].

#### Surgical outcome

In the present series 44.4% (20/45) received resection via the microscopic frontal-keyhole approach and 48.9% (22/45) a median suboccipital telovelar approach. Both approaches offer satisfying anatomical overview and thus facilitate possibility for safe complete tumor removal. Our postoperative complication rate of 20.0% with a shunt-dependency rate of 13.3% highlights the advantages of the keyhole as well as the median suboccipital telovelar approach. 10.0% (2/22) of median suboccipital telovelar approaches and three frontal approaches led to secondary shunt-implantation. 10.0% (1/10) of resected central neurocytoma by a frontal approach led to postoperative ventricle entrapment with secondary cisternostomy. During resection of 4th ventricle lesions intraoperative neuromonitoring was performed to ensure safe functional resection and to reduce cranial nerve lesions. No postoperative wound healing disorders requiring surgical intervention were observed at all.

### Histopathological considerations

IVTs share their predominantly intraventricular location as a result from specific peri- and intraventricular structures from which they arise. The ventricle system raises from telencephalic vesicles from the cranial end of the neural tube as ependymal-lined outpouchings. Into these vesicles the choroid plexus develops from an invagination of primitive pia, creating the choroidal fissure. The epithelium is composed of ependymal cells, origin of ependymomas. Subjacent to the ependymal lining is a subependymal plate of glial cells, from which subependymomas upraise. Residual neuronal precursor cells are found, inter alia, at the septum pellucidum, from which the central neurocytoma may arise [30].

#### Ependymoma

Ependymomas account for 1–5% of intracranial central nervous neoplasms [31]. Arising from the ependymal cells of the ventricular wall, they can be found anywhere along the ventricular system and/or the spinal cord. Intracranially they show predominant occurrence in the posterior fossa e.g. the fourth ventricle (60%) compared to supratentorial location (40%) [1, 14, 32, 33]. Only few case reports on extra-axial ependymomas exist (IEAE). Due to the rare entity of IEAE, no precise relation to intra-axial ependymomas can be made, but the vast majority are intra-axial lesions [34–36].

**Fig. 2:**
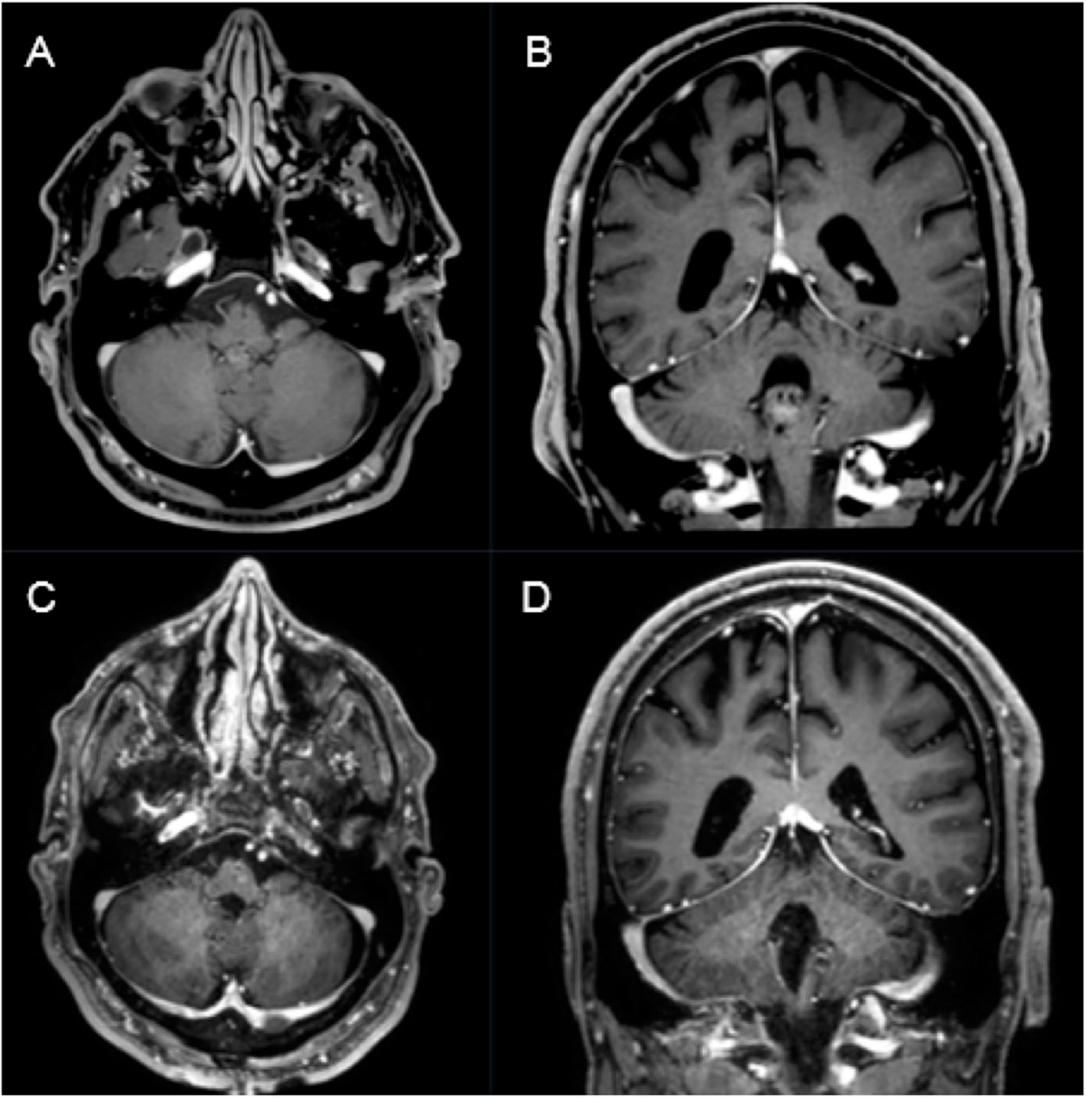
A 27-year-old female patient presented with slight headache and intermitting vertigo. Preoperative T1-weighted gadolinium enhanced MRI showing a heterogeneously enhancing intraventricular mass on the ground of the fourth ventricle consistent with an ependymoma (**a,b**). Postoperative T1-weighted gadolinium enhanced MRI showing complete removal of the tumor through a median suboccipital telovelar approach (**c,d**). Pathological findings confirmed WHO grade II ependymoma

Ependymomas can be found at any age (Fig. 2), with a higher proportion of infratentorial lesions in pediatric patients (mean age 6y), compared to supratentorial tumor location (mean age 18–24y) [30, 31].

The treatment of choice is surgical removal of the tumor, gross total resection shows a prognostic role in recurrence-free and overall outcome. Nevertheless, regarding the mostly benign character of the lesion, avoidance of neurological deficits is of utmost importance and special attention has to be paid to the floor of the fourth ventricle – origin of multiple cranial nerve nuclei and/or ascending/descending tracts [14, 37–39].

In the present series, we achieved complete resection in 84,6% (11/13) and 83,3% (10/12) regarding 4th ventricle ependymomas (Fig. 2), which coincides with the results of previous case series [14, 27, 28, 40–44] (Table 4). Rates of gross total resection (82–91% of patients), cranial nerve deficits or shunt dependency differ among the reports of fourth ventricle ependymomas highlighting the complex anatomy of the fourth ventricle and its floor [42] [14]. In our series, 15.4% (2/13) developed a postoperative shunt-dependent hydrocephalus and 7.7% (1/13) a deterioration of functional outcome (KPSS from 90 to 40%) due to hemorrhagic infarction. One patient developed postoperative new cranial nerve deficits (8.3%), representing a satisfying rate compared to previous reports [14, 27, 28, 40–44]. Our findings coincide with prior studies highlighting good response to operative treatment of ependymomas, also in the fourth ventricle [14, 44]. Our higher rate of complete surgical removal was not associated to higher neurological morbidity or mortality [14, 27, 28, 40–44].

**Table 4:** Case series since 2000 of resected fourth ventricle ependymomas (values displayed are restricted to fourth ventricle ependymomas)

Study	Total patients (adults)	Ependymomas (4th ventr.)	Complete removal (GTR/ependymoma)	Cranial nerve deficits	Mortality
**Chai et al.** [40]	27	13	46.2% (6/13)	–	0/27
**Rajesh et al.** [41]	15	1	0% (0/1)	–	0/1
**Zaheer et al.** [28]	20	2	50.0% (1/2)	0%	0/2
**El-Bahy et al.** [27]	16	4	25.0% (1/4)	50.0%	0/4
Tomasello et al. [42]	45	11	91.0% (10/11)	6.7%	–
Winkler et al. [14]	22	22	82.0% (18/22)	26.0%	0/22
Gök et al. [43]	21	5	80.0% (4/5)	20.0%	0/5
Spagnoli et al. [44]	26	26	69.0% (18/26)	–	1/26
Aftahy et al. (present series)	45	12	83.3% (10/12)	8.3%	0/7

According to the recent 2018 EANO guidelines for diagnosis and treatment of ependymal tumors [45], the role of postoperative radiotherapy in patients with WHO grade II ependymoma undergoing complete removal is still controversial [46, 47]. Two larger retrospective analyses could not find any significant association between radiotherapy and survival outcome [45, 48, 49].

Regarding patients with anaplastic WHO° III ependymoma or incomplete resection of WHO°II ependymomas, adjuvant radiotherapy is recommended [50]. In 2006, Combs et al. described non-inferiority regarding recurrence free survival of fractionated stereotactic radiotherapy (FSRT) compared to conventional radiotherapy, especially at the field borders [51], opposing earlier paradigms in radiotherapy [52].

In our series, two patients underwent postoperative radiotherapy after complete removal. One of them was an anaplastic WHO° III ependymoma, following the actual guidelines of adjuvant therapy. The second case, in 2011, was a tanycytic ependymoma WHO°II, in which the decision for adjuvant radiotherapy was based on ambiguous histopathological findings.

#### Subependymoma

**Fig. 3:**
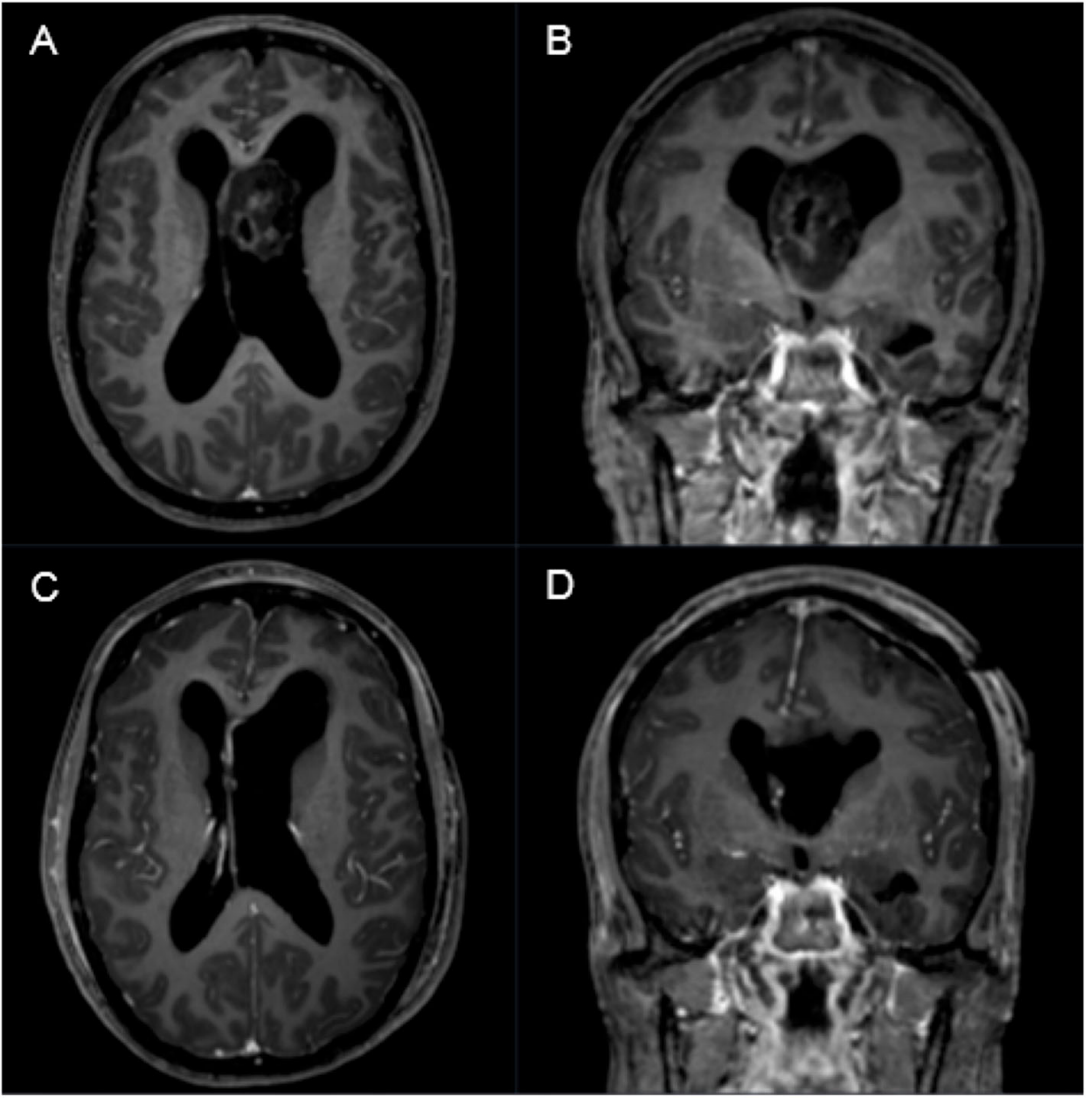
A 44-year-old female patient presented with aggravating headache, vertigo, gait disturbance and concentration disorder in sense of hydrocephalic symptoms for 3–4 months. **a**, **b** Preoperative T1-weighted gadolinium enhanced MRI showing a low to intermediate heterogeneously enhancing intraventricular mass in the third ventricle with consecutive secondary hydrocephalus. **c**, **d** Postoperative T1-weighted gadolinium enhanced MRI showing complete removal of the tumor through a left frontal precoronary transventricular keyhole approach. Pathological findings confirmed WHO grade I subependymoma

Subependymomas account for 0.2–0.7% of intracranial neoplasms [1]. About 82% of subependymomas occur in patients older than 15 years and they show a male predominance (Fig. 3). Subependymomas are generally incidental findings, located in the walls of the fourth (66–70%) and lateral ventricles (30–40%) [53–55]. The foramen of Monro and spinal cord may also be affected [56].

Tumor location and extend of resection are the most important prognostic factors as recurrence has only been reported in case of subtotal resection [53, 57]. Their growth rate tends to be slow [30, 53]. Rarely, aggressive tumors invading brain parenchyma or showing CSF dissemination are described as well [58]. As MR findings of subependymomas are very heterogenous histological confirmation of the diagnosis is mandatory due to several differential diagnosis including ependymomas and central neurocytomas. Thus, a “watch and wait” strategy with regular MRI follow-ups can lead to undertreatment in case of more aggressive entities mistaken for a subependymoma.

Most published reports on subependymomas represent smaller and retrospective cohorts [2, 57–65]. The largest report of Elisabeth Rushing et al. comprised 83 cases, but focused on histopathological findings and does not consider surgical aspects [2].

We report on 21 patients, representing the second largest “surgical” series published to date (Table 5) [2, 57–65]. As radiation or systemic treatment do not apply for subependymomas, surgery remains the only viable option in this entity. The surgical strategy focusses on maximal but safe resection, resulting in permanent absence of the tumor. In the majority of reports, gross total resection could be achieved in > 70% of patients, with low rates of mortality and morbidity [2, 57–65]. In our cohort, we were able to achieve gross total resection in all cases (21/21) with a surgery-related mortality rate of 4,8%.

**Table 5:** Case series since 2000 of resected subependymomas

Study	Patients	Location	Complete removal	Recurrence	Mortality
Nishio et al. [59]	4	Lateral ventricle.	75.0%	0	0
Im et al. [60]	7	Lateral ventricle (6) 3rd ventricle (1)	71.0%	29.0%	0
Mallik et al. [61]	5	3rd ventricle (1) 4th ventricle (4)	n.m.	50.0%	20.0%
Ragel et al. [58]	8	Lateral ventricle (2) 4th ventricle (3) Supratentorial lobar (2) Spinal cord (1)	100%	0	0
Rushing et al. [2]	34%	Lateral ventricle (17) 4th ventricle (15) Others n.m.	53.0%	n.m.	18%
Limaiem et al. [62]	6	Lateral ventricle (5) 4th ventricle (1)	83.3%	0	0
Fujisawa et al. [63]	5	Lateral ventricle (5)	100%	0	0
Kandenwein et al. [57]	11	Lateral ventricle (4) 4th ventricle (7)	73.0%	9.0%	0
Varma et al. [64]	13	Lateral ventricle (5) 3rd ventricle (1) 4th ventricle (8)	92.3%	0	0
Hou Z et al. [65]	26	Lateral ventricle (26)	85.0%	0	3.8%
Aftahy et al. (present series)	21	Lateral ventricle (11) 4th ventricle (10)	100%	0	4.8%

#### Central Neurocytoma

The central neurocytoma is a rare brain tumor with a frequency of 0.1–0.5% of all intracranial central nervous tumors. It is a benign WHO grade II tumor with a 5-year survival of 89% [1, 66, 67]. The origin of these tumors remains unclear, but cell-culture investigations proclaim origin from bipotential progenitor cells that are capable of both neuronal and glial differentiation [4, 68].

**Fig. 4:**
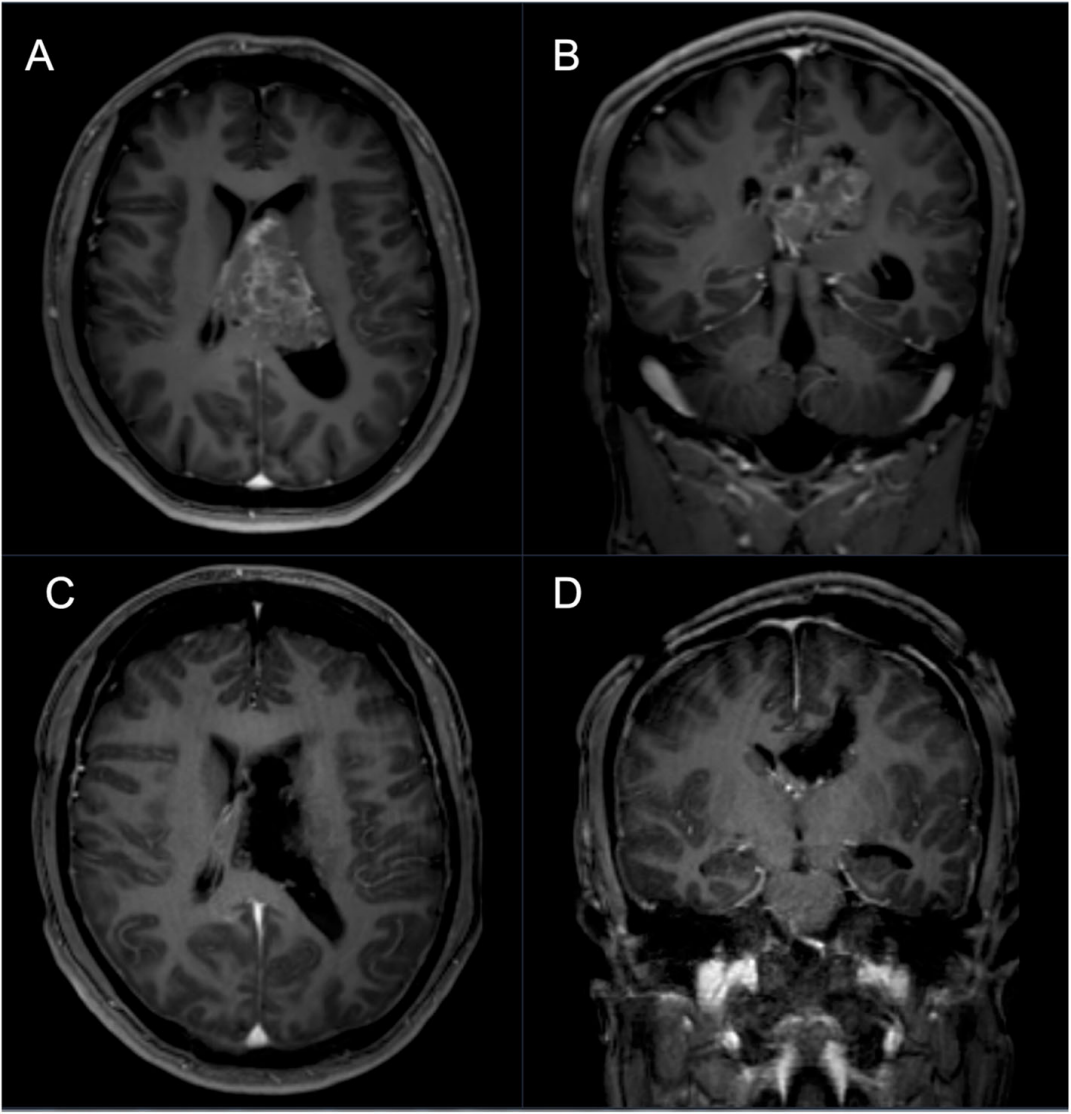
A 32-year-old male patient presented with severe headache, diplopia, vertigo, nausea and a right sided hemiparesis for 2 weeks. **a**, **b** Central neurocytomas appear slightly hypo-intense to iso-intense on T1-weighted and iso-intense to hyper-intense on T2-weighted MRI (hypointensity can indicate the presence of a hemorrhage, cyst, or calcification). Typically, moderate gadolinium enhancement is seen. **c**, **d** Postoperative T1-weighted gadolinium enhanced MRI showing complete removal of the tumor via a left frontal precoronary transcortical keyhole approach. Pathologicalfindings confirmed WHO grade II central neurocytoma

They are typically located in lateral ventricles and/or the third ventricle (Fig. 4). The anterior portion of one lateral ventricle is the most frequent site (50%), followed by combined involvement of the lateral and third ventricles (15%) and the involvement of both lateral ventricles (13%). Surgical resection is primary treatment for central neurocytomas but may also include radiation or systemic therapy. Extend of resection correlates with the rate of recurrence. Patients undergoing subtotal resection are commonly treated with adjuvant stereotactic radiation therapy, resulting in improved outcome compared to surgery alone [69].

Extraventricular neurocytomas are also described and occur in the brain parenchyma, cerebellum or spinal cord [70]. The term “central neurocytoma” is related to the ventricular system.

The tumor’s rarity makes it challenging to define treatment standards. Most institutions, including our center, regrade surgical gross total resection as gold standard for treatment of central neurocytomas with complete removal rates of 30–50% [70–73]. In this cohort, we achieved gross total resection in 90.0% (9/10) [72, 74–76] (Table 6).

**Table 6:** Major case series since 2000 of multimodal treated central neurocytomas (*N* < 10). Note the different treatment options and outcome findings

Study	Patients	Treatments	Complete removal	Outcome	Mortality
Sharma et al. [74]	20	GTR 70.0% (14) + RT STR 30.0% (6) + RT	70.0% (14)	15/20 OS: 66.7%	25%
Leenstra et al. [75]	45	GTR (15) STR (14) GTR/RT (4) STR/RT (7) GTR/RT/CH (2) STR/RT/CH (1) Bx/RT (2)	46.6% (21)	10y OS: 83.0% 10y LC: 60.0%	n.m.
Hallock et al. [76]	20	GTR (10) STR (8) STR/RT (1) No treatment (1)	50.0% (10)	10y OS: 82.0% 10y LC: 61.0%	n.m.
Imber et al. [72]	28	GTR (8) STR (16) GTR/EBRT (1) STR/RT (3)	32.1% (9)	5y PFS: 40.0% 5y PFS: 53.0% n.m. 5y PFS: 67.0%	5y OS: 96.0%, 10y OS: 82.0%
Aftahy et al. (present series)	10	GTR (9) STR (1)	90.0% (9/10)	2y OS: 100%	0%

GTR: gross total resection; STR: subtotal resection; RT: radiotherapy; CH: chemotherapy; EBRT: external beam radiotherapy; Bx: biopsy; OS: overall survival; PFS: progression free survival; LC: local control; SD: standard deviation

After total resection, a five-year survival rate of 99% is reported [70, 77–80], compared to 86% in cases of subtotal resection [79]. Nevertheless, they do not emphasize on adjuvant therapy strategies. This is backed by a pooled analysis by Rades with over 400 cases, which demonstrates superiority of gross total resection regarding overall survival [71]. However, this is in contrast to a series of 45 central neurocytomas showing no significant difference in local tumor control or survival comparing complete and incomplete resection including adjuvant therapy [75]. A systematic review by Garcia et al. displayed that extent of resection was not predictive regarding improved local control [81], while a prospective multi-center study reported that in 71 patients, those with subtotal resection had a 3,8 times higher risk of recurrence [82]. The role of gross total resection remains ambiguous, with several treatment pathways including external beam radiation therapy, stereotactic radiosurgery, re-operation and/or chemotherapy [72]. Whether pathological subtypes or molecular patterns of central neurocytomas play a role in the course of disease and should guide therapy strategies remains .Table 6 highlights the multimodal treatment options of different institutions and their different outcomes [72, 74–76]. Hallock et al. confirmed that gross total resection can be associated with durable long-term outcome and should be first line therapy, but also reported that subtotal resection with no further adjuvant treatment can be seen as salvage treatment with surgery or radiation at the time of clinical and radiographic progression. Nevertheless, he also reported a recurrence rate of about 33% with majority of recurrences within 2.5 years of surgery [76].

Other studies advocated postoperative adjuvant radiotherapy for improved local control of central neurocytomas, but radiation related adverse events of > 60% should be taken into careful consideration [83, 84]. Nevertheless, stereotactic radiosurgery shows promising results in a report of recurrent or residual neurocytomas [85].

Based on our findings we recommend safe, gross total resection to be the first line therapy. In case of recurrence, individual decisions according to overall patient’s condition, tumor location, age and patient’s preference should guide the mode of therapy.

#### Glioependymal cyst (GEC)

The etiology of GECs remains controversial as actual theories on its natural history fail to demonstrate why those cysts occur in different anatomical locations and also do not explain the histological variability in the cyst wall [86]. They are counted to congenital benign lesions with a neuroectodermal origin that share many radiological characteristics with other neuroepithelial lesions. Diagnosis of GECs is confirmed by histological examination [87]. Yasaragil et al. proclaimed that GECs could originate from the tela choroidea migrated somehow during embryogenesis towards brain parenchyma or subarachnoid place [88], resulting in various tumor locations. A systemic review highlighted the difficulties of grouping GECs as few case reports and series are published. Treatment of GECs is indicated if they become symptomatic, whereas surgical approach and goal should be tailored on the individual’s condition [87]. Our case was confirmed by histopathological examination. Surgical decision for complete resection of the cystic tumorous intraventricular lesion was based on the fact that the young, 18 years old patient was admitted due to an acute loss of consciousness and MRI revealed a symptomatic hydrocephalus caused by the lesion. Intraoperatively, the cyst was fenestrated but also tumorous suspicious tissue was detected sticked to the caudate head together with the cyst membrane. As the intraoperative findings were not clear, decision was made to completely resect the cystic lesion. This individual surgical decision was tailored on the current condition and situation (young patient, acute symptoms, suspicious intraoperative findings, possible and feasible complete resection).

#### Study limitations

Our study harbors several flaws and limitations. As it is a retrospective case series, causalities are not possible to draw with respect to clinical outcome. Nevertheless, detailed clinical examination including scores on functional performance as well as a standardized follow up protocol based on a certified neurooncological board are implemented in our clinical workflow. Given the rarity of these lesions prospective inclusion and follow up is hard to achieve within a reasonable time period. Having this in mind, even though we report a relatively large single center series, the absolute amount of cases does not allow for proper statistical analysis. We recommend that, multi-center studies should be conducted to address this problem. In our study we do not focus on long term outcome of different tumor entities, but more on the surgical approach and perioperative outcome. If one wants to address therapy strategies in a whole of these rare lesions, further histopathological, molecular and gene markers have to be taken into account to guide individual therapy strategies. Another problem in rare surgical entities is reflected by the changing therapy modalities, that may bias the therapy outcome, reflected by a learning curve of treating surgeons, various surgeons involved in the treatment or changes in surgical technique. Therefore, in our cohort we limit the report on classic microsurgical approaches. The role of intraventricular endoscopy is not reflected in our series. Even more, local tumor treating strategies like local drug perfusion catheters or laser interstitial thermal therapy might become more important in the future treatment of deep located lesions. Besides its retrospective nature, the analysed patient collective suffers from certain heterogeneities. The different types of IVTs may create inhomogeneity. The number of patients with central neurocytomas and GEC was limited to 10 and 1, respectively, out of 45 total patients, which could lead to variability in the results. We included them in the analysis because all aspects of IVTs should be reflected and because basic surgical techniques are similar for IVTs.

## Conclusion

Our surgical findings emphasize safe complete resection throughout all above analyzed neuroepithelial lesions. Surgical treatment can remain both safe and feasible, if institutional experience is given. Satisfying long-term survival and also cure is possible by complete removal. Regarding complete resected subependymomas shorter follow-up can be discussed, too. It should be noted, that gross total resection should always be performed under functional improving aspects due to mostly benign nature of IVTs. Further data is needed to evaluate standard of care and alternative therapy options in rare cases of tumor recurrence or in case of patient collective not suitable for operative resection.

## Data Availability

The datasets used and/or analysed during the current study are available from the corresponding author on reasonable request.

## Abbreviations

CDG: Clavien Dindo Scale
CSF: Cerebrospinal Fluid
FSRT: Fractionated Stereotactic Radiotherapy
GEC: Glioependymal cyst
IEAE: Extra-axial ependymomas exist
IVT: Intraventricular neuroepithelial tumors
KPSS: Karnofsky Performance Status Scale
MRI: Magnet Resonance Imaging
WHO: World Health Organization
